# Impaired Fibrinolysis in Patients with Isolated Aortic Stenosis is Associated with Enhanced Oxidative Stress

**DOI:** 10.3390/jcm9062002

**Published:** 2020-06-25

**Authors:** Jakub Siudut, Joanna Natorska, Ewa Wypasek, Łukasz Wiewiórka, Elżbieta Ostrowska-Kaim, Sylwia Wiśniowska-Śmiałek, Krzysztof Plens, Jacek Legutko, Anetta Undas

**Affiliations:** 1 Institute of Cardiology, Jagiellonian University Medical College, 31-202 Krakow, Poland; jakub.siudut@doctoral.uj.edu.pl (J.S.); j.natorska@szpitaljp2.krakow.pl (J.N.); j.legutko@szpitaljp2.krakow.pl (J.L.); 2 Krakow Center for Medical Research and Technologies, John Paul II Hospital, 31-202 Krakow, Poland; 3 Faculty of Medicine and Health Sciences, Andrzej Frycz Modrzewski Krakow University, 30-705 Krakow, Poland; e.wypasek@szpitaljp2.krakow.pl; 4 Department of Interventional Cardiology, John Paul II Hospital, 31-202 Krakow, Poland; Drlucwie@gmail.com (Ł.W.); elaostr@gmail.com (E.O.-K.); 5 Department of Cardiac and Vascular Diseases, John Paul II Hospital, 31-202 Krakow, Poland; swisniowskasmialek@gmail.com; 6 KCRI, 30-055 Krakow, Poland; plens_krzysztof@o2.pl

**Keywords:** aortic stenosis, oxidative stress, fibrinolysis, α2-antiplasmin, PAI-1

## Abstract

Aortic stenosis (AS) has been associated with impaired fibrinolysis and increased oxidative stress. This study aimed to investigate whether oxidative stress could alter fibrin clot properties in AS. We studied 173 non-diabetic patients, aged 51–79 years, with isolated AS. We measured plasma protein carbonylation (PC) and thiobarbituric acid reactive substances (TBARS), along with plasma clot permeability (K_s_), thrombin generation, and fibrinolytic efficiency, which were evaluated by two assays: clot lysis time (CLT) and lysis time (Lys50). Coagulation factors and fibrinolytic proteins were also determined. Plasma PC showed an association with AS severity, reflected by the aortic valve area and the mean and maximum aortic gradients. Plasma PC was positively correlated with CLT, Lys50, plasminogen activator inhibitor-1 (PAI-1), and tissue factor (TF) antigens. TBARS were positively correlated with maximum aortic gradient, Lys50, and TF antigen. Regression analysis showed that PC predicted prolonged CLT (>104 min; odds ratio (OR) 6.41, 95% confidence interval (CI) 2.58–17.83, *p* < 0.001) and Lys50 (>565 s; OR 5.83, 95% CI 2.23–15.21, *p* < 0.001). Multivariate regression analysis showed that mean aortic gradient, PC, α2-antiplasmin, PAI-1, and triglycerides were predictors of prolonged CLT, while PC, α2-antiplasmin, and fibrinogen were predictors of Lys50. Our findings suggest that elevated oxidative stress contributes to impaired fibrinolysis in AS and is associated with AS severity.

## 1. Introduction

Aortic stenosis (AS) is the most common valvular heart disease in high income countries. The prevalence of AS increases with age and it affects about 0.4% of the general population, 1.3% of the population that is over 60 years old, and up to 3.9% in septuagenarians and elder patients [1,2]. Nowadays, AS is defined as an active and multifaceted disease that shares many similarities with atherosclerosis [3]. The pathobiology of degenerative AS involves interrelated complex processes such as inflammation, oxidative stress, angiogenesis, fibrosis, and osteogenic differentiation. Recent studies have shown the essential role of oxidative processes in the pathogenesis of AS [4,5,6]. Low density lipoproteins (LDL) and lipoprotein(a) (Lp(a)) have been reported to infiltrate through the damaged endothelium and into the fibrosa and promote the recruitment of inflammatory cells into the aortic valve. The presence of reactive oxygen species (ROS) contributes to increased lipid oxidation [3,7]. In vitro studies have shown that oxidized LDL (Ox-LDL) and Lp(a) (Ox-PL) not only promote osteogenic differentiation and calcification in valvular cells, resulting in faster AS progression, but also induce a pro-oxidant state [8,9,10].

It has been shown that an enhanced oxidative state affects not only lipoproteins but also plasma proteins, including those involved in blood coagulation and fibrinolysis. It has been demonstrated that fibrinogen, coagulation factors V, VIII, X, XIII, and fibrinolysis proteins including tissue plasminogen activator (tPA) are sensitive to ROS [11]. The most common and irreversible protein modification induced by ROS is carbonylation, which affects specific amino acids in the side chains, e.g., arginine, threonine, proline, or lysine, and eventually may alter protein conformation, activity, and function [12]. Fibrinogen purified from patients with myocardial infarction (MI) has been shown to have over three-fold increased carbonylation in comparison with healthy subjects, and it has been positively associated with total plasma protein carbonyls (PC) and thiobarbituric acid reactive substances (TBARS) [13]. Moreover, carbonylated fibrinogen has been reported to alter the kinetics of fibrin formation and clot structure, along with biomechanical fibrin properties [12]. Increased plasma carbonylation has also been demonstrated in patients with type 2 diabetes as a strong predictor of reduced fibrinolytic efficiency, independent from plasminogen activator inhibitor-1 (PAI-1) [14].

Several studies have suggested that fibrin, the final product of blood coagulation initiated by the tissue factor (TF), is involved in the pathogenesis of AS [15,16]. Natorska et al. have shown that the area of TF expression co-localizes with the areas of fibrin deposits within human stenotic valve leaflets, suggesting that the conversion of fibrinogen into fibrin takes place within the stenotic valves [15]. The amount of fibrin within the stenotic valves, as well as the pressure gradients across the aortic valve, have been positively correlated with prolonged plasma fibrin clot lysis time in severe AS patients, indicating impaired fibrinolysis [17]. It is still unclear whether hypofibrinolysis is a cause or consequence of AS, though it has been speculated that defective enzymatic fibrin degradation in some individuals might predispose them to faster progression of this disease [18]. Factors influencing blood coagulation and fibrinolysis, such as oxidation, may affect the signaling pathways connected with aortic valve inflammation and calcification [4,19]. It is unclear whether disturbed hemostasis, particularly the prothrombotic fibrin clot phenotype and hypofibrinolysis, is related to enhanced oxidative stress in AS.

The aim of our study was to investigate whether oxidative stress affects fibrin clot properties in AS without significant atherosclerotic disease.

## 2. Materials and Methods

We enrolled 173 consecutive patients, aged from 18 to 80 years, with isolated AS, which was diagnosed based on the criteria proposed by the European Association of Echocardiography [20]. Transthoracic echocardiography was performed using a Philips iE33 (Philips Electronics, Andover, MA, USA) imaging device in accordance with the European Society of Cardiology (ESC) guidelines [20]. The aortic valve area (AVA) was calculated using the standard continuity equation. The transvalvular gradient was measured by Doppler echocardiography, using the modified Bernoulli equation. A congenital bicuspid aortic valve (BAV) was diagnosed either by echocardiography or by direct visualization at the time of surgery, in patients undergoing valve replacement.

The following exclusion criteria were used: rheumatic AS; atrial fibrillation; acute infection, including endocarditis; previous cardiac surgery or requirement for mitral valve surgery; history of myocardial infarction or stroke; documented advanced atherosclerosis, including epicardial artery stenosis (>20% diameter on coronary angiography performed within the preceding month); carotid artery stenosis (>30% diameter observed on ultrasound examination) or peripheral artery disease (subjects with intermittent claudication or ankle brachial index < 0.9); diagnosed malignancy; end-stage kidney disease (stage 5); diabetes; and pregnancy. Obesity was defined as body mass index (BMI) ≥ 30 kg/m^2^. Hyperlipidemia was diagnosed based on either a total cholesterol level of 5.2 mmol/L or more or statin therapy. Arterial hypertension was diagnosed based on a history of hypertension (blood pressure > 140/90 mm Hg) or pre-admission antihypertensive treatment. Chronic obstructive pulmonary disease (COPD) was diagnosed based on the signs and symptoms and the results of spirometry. Chronic kidney disease was defined as an estimated glomerular filtration rate of less than 60 mL/min per 1.73 m^2^.

The study was approved by the Local Ethical Committee. All participants provided informed consent in accordance with the Declaration of Helsinki.

### 2.1. Laboratory Investigations

Blood samples were drawn from antecubital veins, with minimal stasis. The complete blood cell count, lipid profile, glucose, and creatinine were assayed by routine laboratory techniques. High-sensitivity C-reactive protein (hsCRP) and Lp(a) were determined using immunoturbidimetry (Roche Diagnostics, Mannheim, Germany). Fibrinogen was measured using the Clauss method (Multifibren U, Siemens Healthcare, Marburg, Germany). Factor II activity was determined using a coagulometric method (Thromborel S, Siemens Healthcare). Plasma α2-antiplasmin and plasminogen were measured by chromogenic assays (Siemens Healthcare). Commercially available ELISA tests were used to determine TF antigen (Zymutest Total Tissue Factor Antigen), PAI-1 antigen (Zymutest PAI-1 Antigen), and thrombin activatable fibrinolysis inhibitor (TAFI) zymogen (Zymutest Activatable TAFI, all Hyphen BioMed, Neuville-Sur-Oise, France).

To evaluate fibrin clot properties and thrombin generation, blood samples were mixed with 3.2% sodium citrate (vol/vol, 9:1) and centrifuged at 2000 × *g* for 10 min within 30 min since the draw. The supernatant was aliquoted and stored at −80 °C until further analysis. All measurements were performed by technicians who were blind to the origin of the samples.

### 2.2. Clot Permeability

Thrombin-based fibrin clot permeation was measured as described previously [21]. Briefly, citrated plasma samples were mixed with 20 mmol/L calcium chloride and 1 U/mL human thrombin (Calbiochem, San Diego, CA, USA). In the second method, TF-based fibrin clot permeability [22], citrated plasma samples were mixed with 10 pmol/L TF (Innovin, Siemens Healthcare Diagnostics), 4 μmol/L phospholipids (Rossix AB, Mölndal, Sweden), and 20 mmol/L CaCl_2_. Mixtures were immediately added to a plastic tube. After incubation in a wet chamber, tubes containing the clots were connected to a reservoir containing Tris buffer saline. The volume flowing through the gels was measured within 60 min. A permeation coefficient (K_s_), which indicates the pore size, was calculated from the equation: K_s_ = Q × L × η/t × A × Δp, where Q is the flow rate in time t; L is the length of a fibrin gel; η is the viscosity of liquid (in poise); t is percolating time; A is the cross-sectional area (in cm^2^); and Δp is a differential pressure (in dyne/cm^2^). The intra- and inter-assay coefficients of variation were 5–7%. The reference range for the healthy controls was 4.5–11.7 × 10^−9^ cm^2^ for the thrombin-based K_s_ and 5.2–10.0 × 10^−9^ cm^2^ for the TF-based K_s_.

### 2.3. Scanning Electron Microscopy (SEM)

After the permeability measurement, fibrin clots were fixated in 2.5% glutaraldehyde (in 0.1 M phosphate-buffered saline solution, pH 7.4) for 2 h. Fixed clots were gently removed from tubes, dehydrated in graded ethanol solutions, dried by the critical point procedure, and sputter coated with gold. Finally, the treated clots were scanned in ten different areas (microscope JEOL JCM6000; JEOL Ltd., Tokyo, Japan). We analyzed fibrin clots from 20 randomly selected AS patients with similar fibrinogen levels, including 10 with the highest protein carbonyl (PC) content (4th quartile, >3.05 nmol/mg) and 10 with lower PC levels (1st–3rd quartiles). For each clot, 4 micrographs were performed at a machine magnification of 10,000 × in order to evaluate fibrin diameter based on 40–50 fibers per clot, using ImageJ software (US National Institutes of Health, Bethesda, MD, USA).

### 2.4. Plasma Clot Lysis Assays

To assess fibrinolytic efficiency, two plasma-based assays were used. Firstly, clot lysis time (CLT) was measured, as previously described by Pieters et al. [23]. Briefly, citrated plasma was mixed with 15 mmol/L calcium chloride, 0.5 U/mL human thrombin (Calbiochem), 10 μM phospholipid vesicles (Rossix), and 18 ng/mL recombinant tPA (rtPA; Boehringer Ingelheim, Ingelheim, Germany). The mixture was transferred to a microtiter plate and its turbidity was measured at 405 nm at 37 °C using the microplate reader (Tecan Sunrise, Maennedorf, Switzerland). CLT was defined as the time from the midpoint of the clear-to-maximum-turbid transition, which represents clot formation, to the midpoint of the maximum-turbid-to-clear transition. Secondly, lysis time (Lys50) was measured, as previously described by Carter et al. [24]. Briefly, citrated plasma was mixed with 7.5 mmol/L calcium chloride, 0.03 U/mL human thrombin (Calbiochem), and 12.5 ng of rtPA (Boehringer Ingelheim). The mixture was added to each well of a microtiter plate. The turbidity was measured at 340 nm at 37 °C using the microplate reader (Tecan Sunrise). Lys50 was calculated as the time from full clot formation to the time when absorbance was reduced by 50%. The intra- and inter-assay coefficients of variation were 6–8%. The 95% reference range assessed in our lab for the healthy controls was 54.8–122.4 min for CLT and 257–598 s for Lys50.

### 2.5. Thrombin Generation

Thrombin generation kinetics was measured with the Calibrated Automated Thrombogram (CAT) (Thrombinoscope BV, Maastricht, the Netherlands), according to the manufacturer’s instructions, in a 96-well plate fluorometer (Ascent Reader, Thermolabsystems OY, Helsinki, Finland). Briefly, 80 µL of platelet-poor plasma was diluted with 20 µL of the reagent containing 5 pmol/L recombinant TF, 4 µmol/L phosphatidylserine/phosphatidylcholine/phosphatidylethanolamine vesicles, and 20 µL of FluCa solution (Hepes, pH 7.35, 100 mmol/L CaCl_2_, 60 mg/mL bovine albumin, and 2.5 mmol/L Z-Gly-Gly-Arg-amido methyl coumarin). Each plasma sample was analyzed in duplicate, and the intraassay variability was 7.2%. The maximum concentration of thrombin formed during the recording time is described as “Peak thrombin”, and the area under the curve represents endogenous thrombin potential (ETP). The “lag time” is the time from the start of analysis until thrombin starts to generate.

### 2.6. Plasma Carbonyl Content and Malondialdehyde Levels in Plasma

The oxidative modification of the plasma proteins was assessed based on carbonyl content, using 2,4-dinitrophenylhydrazine (DNPH; AppliChem, Darmstadt, Germany), as reported previously [25]. Briefly, DNPH reacts with protein carbonyls (PC), forming a Schiff base to form the corresponding hydrazone. The absorbance was read spectrophotometrically at 370 nm using the microplate reader (Tecan Sunrise). The reference range for healthy subjects in our laboratory was 0.54–2.03 nmol/mg. TBARS is an assay to evaluate lipid peroxidation by measuring the malonaldehyde levels generated during the oxidative degradation of lipids. The plasma TBARS levels were measured using a commercially available assay kit (Cayman Chemical, Ann Arbor, MI, USA) in accordance with the manufacturer’s instructions.

### 2.7. Statistical Analysis

We decided to use quartile-based analysis to find the determinants of the longest lysis times in AS patients, including global oxidative biomarkers. The fourth quartile of CLT in AS patients corresponds to the 90th percentile of CLT in apparently healthy subjects (106 min). The study was powered to have an 80% chance of detecting a 10% difference in the total PC, using a significance level of 0.05, based on the values of total PC in the previous study [13]. To demonstrate such a difference or greater, 30 patients or more in the top quartile group (±30% attrition rate) were required.

Categorical variables were presented as numbers and percentages. Continuous variables were expressed as mean ± standard deviation or median and interquartile range. Normality was assessed by the Shapiro–Wilk test. Equality of variances was assessed using the Levene’s test. Differences between groups were compared using Student’s or Welch’s *t*-test, depending on the equality of variances for normally distributed variables. The Mann–Whitney *U*-test was used for non-normally distributed continuous variables. Categorical variables were compared by the Pearson chi-squared test or Fisher’s exact test. Pearson’s correlation coefficient was calculated for normally distributed variables while Spearman’s correlation coefficient was determined for non-normally distributed variables. To investigate the associations between lysis time and both demographic and laboratory parameters, simple and multiple linear regression analyses were performed. Normal distribution of residuals was checked using the Shapiro–Wilk test. Multiple linear regression analyses were performed to investigate the associations between the CLT and both echocardiographic and laboratory parameters. Adjusted R^2^ was reported for linear regression models. Determinants of the longest CLT and Lys50 (top quartile) were determined by univariate and multivariate logistic regression models. The multivariate models were fitted using backward stepwise regression and adjusted for age, sex, and BMI. All statistical analyses were performed with JMP®, Version 14.2.0 (SAS Institute INC., Cary, NC, USA).

## 3. Results

We examined 173 nondiabetic patients with isolated AS, including 84 males (48.6%), with a mean age of 67 years (Table 1). Patients with severe AS predominated (*n* =143, 83.7%). BAV was identified in 64 (37.0%) patients, who were younger in comparison with those with tricuspid aortic valve stenosis (60 (53–68) vs. 69 (63–77) years, *p* < 0.001) and had higher LDL-C levels (2.76 (2.13–3.37) vs. 2.24 (1.90–3.06) mmol/L, *p* = 0.021), with no other differences. Plasma total PC levels were 2.76 ± 0.45 nmol/mg. The TBARS levels were 7.76 (6.78–9.09) nmol/mL. Based on our reference values, both parameters were markedly higher in comparison with healthy subjects. We observed a positive correlation between total PC and TBARS levels (*r* = 0.48, *p* < 0.001). The presence of BAV was not associated with oxidative stress markers.

### 3.1. Oxidation and AS

**Figure 1:**
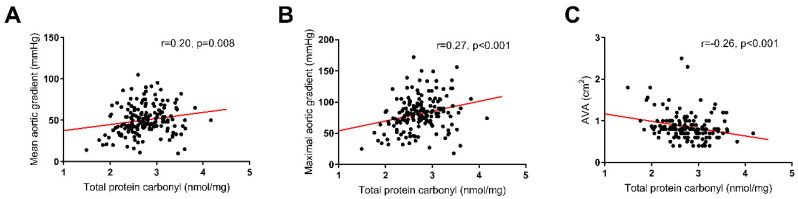
Correlations of the total protein carbonyl levels with the echocardiographic parameters: (**A**) mean aortic gradient; (**B**) maximum aortic gradient; and (**C**) aortic valve area (AVA).

**Figure 2:**
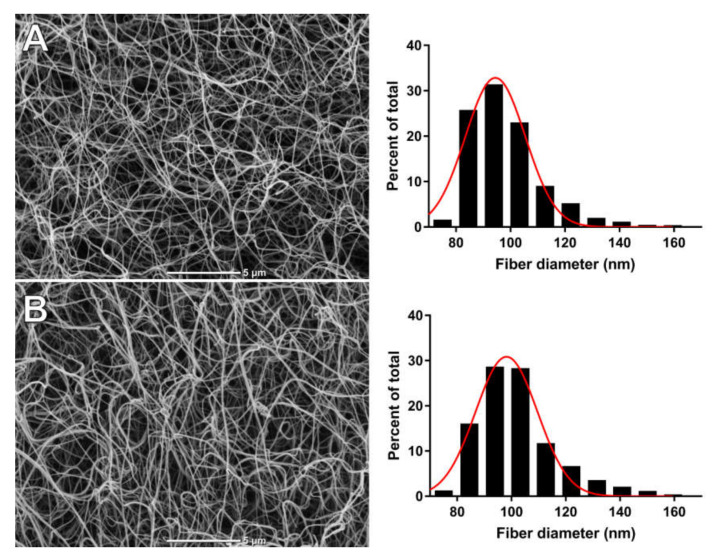
Representative scanning electron microscopy images of fibrin mesh with histograms for fibrin diameters in AS patients with: (**A**) the highest total protein carbonyl level (defined as the top quartile, >3.05 nmol/mg); and (**B**) lower PC levels (<3.05 nmol/mg).

**Figure 3:**
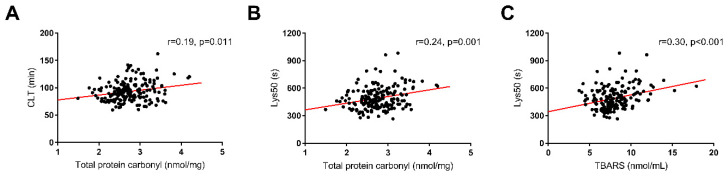
Correlations of oxidative markers levels with fibrinolytic efficiency: (**A**) total protein carbonyl (PC) with clot lysis time (CLT); (**B**) PC with lysis time (Lys50); and (**C**) thiobarbituric acid reactive substances (TBARS) with Lys50.

Total PC was weakly correlated with age (*r* = 0.16, *p* = 0.03) but not with other demographic parameters. The total PC level was correlated with AS severity, as reflected by the AVA (*r* =−0.26, *p* < 0.001) as well as the mean and maximum aortic gradients (*r* = 0.20, *p* = 0.008 and *r* = 0.27, *p* < 0.001, respectively; (Figure 1)). Notably, PC showed a positive correlation with glycemia (*r* = 0.27, *p* < 0.001). Among the hemostatic variables, we observed weak associations of PC solely with PAI-1 (*r* = 0.19, *p* = 0.011) and TF levels (*r* = 0.20, *p* = 0.008) but not with K_s_ as measured with two assays or thrombin generation parameters. Patients with the highest PC levels (defined as the top quartile, >3.05 nmol/mg) had slightly thinner fibrin fibers compared with those with the lowest PC levels (Figure 2, 99.0 ± 3.9 vs. 93.8 ± 5.6 nm, *p* = 0.045). There were positive, though weak, correlations of PC with CLT (*r* = 0.19, *p* = 0.011) and Lys50 (*r* = 0.24, *p* = 0.001) (Figure 3).

There were no correlations between plasma TBARS levels and demographic parameters. TBARS were positively, though weakly, correlated with the maximum aortic gradient (*r* = 0.18, *p* = 0.022) but not with other echocardiographic parameters. Like the total PC, TBARS showed weak associations with glycemia (*r* = 0.25, *p* = 0.001) and TF (*r* = 0.16, *p* = 0.031) but not with PAI-1, K_s_, or thrombin generation. TBARS were positively associated with Lys50 (*r* = 0.30, *p* < 0.001) but not with CLT (Figure 3).

The predictors of high total plasma PC in AS patients (the top quartile), adjusted for age, sex, BMI, and cigarette smoking, were hypertension (OR 3.46, 95% CI 1.07–11.17, *p* = 0.038) and glycemia (OR 2.43, 95%CI 1.43–4.14, *p* = 0.001, per 1 mmol/L). The only predictor of plasma high TBARS (the top quartile, >9.03 nmol/mL), adjusted for age, sex, BMI, and cigarette smoking, was glycemia (OR 1.93, 95% CI 1.16–3.19, *p* = 0.011, per 1 mmol/L).

### 3.2. Hypofibrinolysis in AS Patients

Patients with prolonged CLT (*n* = 43), defined as a value above 104 min (top quartile), were characterized by a higher BMI (+16.7%), both mean and maximum aortic gradients (+27.4% and +26.4%, respectively), plasma PC (+10.5%), prothrombin (+7.2%), α2-antiplasmin (+4.3%), plasminogen (+7.4%), PAI-1 (+99%), total cholesterol (+6.5%), and triglycerides (+52.4%, all *p* <0.01), as compared with the remaining patients (Table 1). The differences remained significant after adjustment for age, sex, and BMI, except prothrombin levels (*p* = 0.086). We did not observe CLT-related differences in K_s_.

In our study group, CLT was positively correlated with mean aortic gradient (*r* = 0.23, *p* = 0.002) but not with other echocardiographic parameters. CLT showed the strongest linear correlations with prothrombin (*r* = 0.41, *p* < 0.001), PAI-1 (*r* = 0.56, *p* < 0.001), plasminogen (*r* = 0.38, *p* < 0.001), and α2-antiplasmin (*r* = 0.40, *p* < 0.001). Lys50 correlated weakly with AVA (*r* = −0.16, *p* = 0.048) but not with either aortic gradient. In contrast to CLT, Lys50 was associated with prothrombin (*r* = 0.37, *p* < 0.001) and α2-antiplasmin (*r* = 0.33, *p* < 0.001) but not with PAI-1 or plasminogen. Moreover, both the mean and maximum aortic gradients were positively correlated with plasma PAI-1 levels (both *r* = 0.18, *p* = 0.019).

BAV patients, who were 9 years younger than the remainder, had shorter Lys50 (445 (373–516) vs. 486 (421–574) s, *p* = 0.002) along with higher plasminogen (102 (93–112)% vs. 95 (87–106)%, *p* = 0.01) and TAFI (84.7 (77.9–88.9)% vs. 78.5 (71.2–88.1)%, *p* = 0.03), while the level of TF was lower (70.4 (59.2–82.5) vs. 84.2 (71.7–94.9) pg/mL, *p* < 0.001). No differences related to BAV were observed for K_s_, CLT, or PAI-1.

### 3.3. Determinants of Prolonged Fibrinolysis

The multiple linear regression analysis, incorporating all significant variables associated with CLT and adjusted for age, sex, and BMI, showed that PAI-1, mean aortic gradient, α2-antiplasmin, prothrombin, and triglyceride levels remained significantly associated with CLT in AS patients (Table 2). Although we did not observe associations between CLT and any of the oxidative markers in the linear regression, we observed them in the logistic regression analysis. After the addition of fibrinolysis confounders into the model, including plasminogen and TAFI, plasma PC, CRP, and lipoprotein(a), only TAFI and CRP were not associated with CLT (Table 2).

Logistic regression analyses, adjusted for age, sex, and BMI, were used in order to ascertain the determinants of the longest CLT and Lys50 (top quartiles). Multivariate analysis showed that mean aortic gradient, plasma PC, α2-antiplasmin, plasminogen, PAI-1, and triglycerides were the predictors of prolonged CLT (Table 3). Prolonged Lys50 was predicted by plasma PC, α2-antiplasmin, and fibrinogen levels (Table 3).

Additional analyses based on comparisons between the four groups of patients, divided according to the median plasma levels (interquartile range) of PC (cut-off 2.72 nmol/mg) and median CLT (cut-off 91.5 min), showed that patients with a higher PC and longer CLT had a higher mean aortic gradient compared to patients with a lower PC and shorter CLT (55 (45–75.5) vs. 43 (36.5–56) mmHg, *p* = 0.003).

## 4. Discussion

To our knowledge, this study is the first to show that increased oxidative stress, as evidenced by elevated plasma PC, is associated with reduced fibrinolytic efficiency and disease severity in patients with isolated AS. Patients with enhanced oxidation had thinner fibrin fibers, which was supported by SEM analysis, indicating changes in the nanostructure of the fibrin networks, despite no effect on clot permeability reflecting the average pore size in the meshworks. This study identifies new predictors of impaired fibrinolysis in AS patients, showing the significant contribution of PC and antiplasmin activity, and highlights the relevance of a prothrombotic and hypofibrinolytic state in AS.

In order to determine the efficiency of fibrinolysis, we performed two plasma-based lysis assays, namely CLT, according to Pieters et al. [23], with a relatively low concentration of tPA (18–20 nmol/L), and the second one, according to Carter et al. [24], with a high tPA concentration (83 nmol/L). This methodological difference might justify why oxidative stress has a crucial effect on impaired fibrinolysis in AS despite the various demographic and laboratory determinants of prolonged lysis time between these assays (Siudut et al. manuscript in review). The assay by Pieters et al. that was introduced in 2019 and supported by the International Society on Thrombosis and Hemostasis Subcommittee has been used in a few studies [23,25,26] and is known to be more sensitive to endogenous PAI-1 levels, especially in both hypofibrinolytic and/or hyperfibrinolytic conditions [27]. On the other hand, the Lys50 assay has been previously applied in studies investigating fibrinolytic capacity in patients with metabolic syndrome, including diabetes mellitus [24]. The clinical trial PLATO, with ticagrelor in patients with acute coronary syndrome, confirmed the clinical relevance of Lys50 by showing that each 50% increase in Lys50 was associated with cardiovascular death or spontaneous myocardial infarction [28]. In AS patients, we observed that the increased carbonylation of plasma proteins is likely to affect fibrinolysis, as demonstrated by the positive associations between plasma PC and both CLT and Lys50. Moreover, the plasma PC level was the determinant of prolonged CLT and Lys50 in AS patients, which is a novel observation that suggests that, in other diseases associated with elevated oxidative stress, including atherosclerosis, PC might also affect fibrinolysis potential. We observed that, regardless of the tPA concentration used in the lysis assay, the mean aortic gradient and AVA were associated with CLT and Lys50, respectively. Due to stronger associations with the markers of AS severity, CLT may be a more appropriate assay to evaluate plasma fibrinolytic potential in this disease.

Looking for the mechanisms underlying hypofibrinolysis in AS, we demonstrated that α2-antiplasmin, the key plasmin inhibitor [29], is a crucial determinant of prolonged lysis time in patients with AS, regardless of the tPA concentration used. All patients had this variable within the normal range; however, small changes had a significant impact on fibrinolysis potential in global assays. Meltzer et al. demonstrated that α2-antiplasmin was associated with CLT to a similar extent to TAFI [30]. Recent studies have shown that elevated circulation α2-antiplasmin levels have been associated with hypofibrinolysis and increased thrombotic risk [31]. Hypothetically, α2-antiplasmin may become a novel therapeutic target to possibly prevent thromboembolism [32,33].

Contrary to our expectations, we did not observe any associations between K_s_ and oxidative markers in AS. However, fibrin clots generated from the plasma of patients with higher levels of PC were composed of slightly thinner fibrin fibers than the clots of patients with lower PC levels. Fibrinogen is the most sensitive to the oxidative modification compared to the other main plasma proteins [34]. There have been several known site-specific oxidative modifications of fibrinogen with different types and effects on fibrin function and clot structure associated with smaller fibrin fibers, higher fibrin density, and prolongation of fibrin clot lysis [35]. Moreover, we evaluated endogenous thrombin generation due to the known impact of thrombin concentration on fibrin structure and function. Previous data have shown that at higher thrombin concentrations fibrin clots are more compact and composed of thinner fibers [36]. However, in the current study, we did not observe such associations, which may result from our evaluation of thrombin generation potential instead of in vivo markers of thrombin generation.

The choice of oxidative stress markers in the current study deserves comment. Firstly, PC is a reaction that introduces reactive carbonyl groups, mainly ketones and aldehydes, into a protein structure [37]. This modification may occur as a result of the direct oxidation of several amino acid residues or glycation of the amino group of lysine. The quantitative detection of protein carbonyls and TBARS in plasma samples is an indirect way to determine the level of oxidative stress. These methods are widely used and accepted irreversible markers of protein oxidation [37,38]. Moreover, since AS and atherosclerosis share numerous pathobiological similarities, including mechanisms of oxidative stress, we decided to determine the same markers of oxidative stress as shown by Becatti et al. [13] in patients with concomitant advanced atherosclerosis. Both oxidative stress and impaired fibrinolysis have been implicated in the pathophysiology of several diseases which are associated with AS, such as coronary artery disease [39,40] and type 2 diabetes [14,41]. To exclude the possibility that enhanced oxidative stress and hypofibrinolysis are attributed to those diseases and not to AS itself, previous myocardial infarction, stroke, and type 2 diabetes were the exclusion criteria in our study. Nevertheless, despite the absence of diabetes, glycemia was the strongest determinant of the oxidative stress in patients with AS. This observation suggests that even a slight increase in the glucose level typical of prediabetes may induce oxidative stress and the carbonylation of plasma proteins.

This study is the first to show that patients with BAV, being the most common congenital valvular heart defect, with a prevalence of 2% in the general population [42], had shorter lysis times and enhanced fibrinolysis as reflected by higher plasminogen and TAFI. The key factor affecting this observation is the much younger age of patients with BAV, as it has been shown that lysis efficiency is inversely associated with age [24]. BAV patients had a similar severity of AS; therefore, we may speculate that impaired lysis, as compared to healthy controls, is a feature of the BAV syndrome associated with various vascular abnormalities. This study suggests that hypofibrinolysis related to higher TAFI might be a component of this vascular syndrome, and this concept warrants further study.

This study had several limitations. Due to the limited number of patients with mild and moderate stages of AS, our observations should not be extrapolated to these patients. Most likely, the impact of oxidation at lower aortic gradients will be weaker. Antioxidant mechanisms were not assessed in this study, since we focused on the net effect of the balance between pro-and antioxidant effects in AS; however, further studies on this issue are needed. We did not determine the activity of fibrinolytic proteins such PAI-1 and TAFI. Moreover, we did not measure antioxidant capacity—an important modifier of oxidative stress, which is usually decreased in the presence of ROS. Furthermore, we did not measure the oxidation of purified proteins, such as fibrinogen or plasminogen, whose oxidative modifications have been shown to modulate fibrin properties [14,43]. It also remains to be established whether AS treatment leads to the normalization of hypofibrinolysis and reduces oxidative stress. On a molecular level, further studies should focus on the identification of the site-specific oxidative modification of fibrinogen molecules, plasminogen, and other types of fibrinolysis-involved proteins underlying the resistance to fibrinolysis that was observed in isolated AS.

To conclude, in our opinion, the global higher oxidation may contribute to more impaired fibrinolysis and ultimately to the progression of AS. Enhanced oxidation in AS patients is probably driven by age and comorbidities (including hypertension and/or prediabetic state). It is also possible that increased shear stress in AS might be involved in enhancing oxidative stress. Our findings might help researchers to better understand the pathophysiology of AS and the role of enhanced oxidative stress. It remains to be established which mechanisms underlie the association between protein oxidation and impaired fibrin clot lysis in AS. It is tempting to speculate that interventions altering fibrin properties and lysability might have therapeutic potential in patients at risk of AS progression.

## Figures and Tables

**Table 1:** Characteristics of patients with aortic stenosis (AS). The top quartile of clot lysis time (CLT) corresponds to 90th percentile of CLT in apparently healthy subjects (106 min).

Variable	Total Cohort (*n* = 173)	CLT ≥ 104 min (*n* = 43)	CLT < 104 min (*n* = 130)	*p*-Value
Age, years	67.0 (59.0–72.5)	67 (57–71)	67 (59–74)	0.40
Male, *n* (%)	84 (48.6)	13 (30.2)	71 (54.6)	0.006
BMI, kg/m^2^	27.4 (24.6–30.8)	30.8 (26.6–32.7)	26.4 (24.1–29.7)	<0.001
Current smoking, *n* (%)	36 (20.8)	8 (18.6)	28 (21.5)	0.68
**Comorbidities, *n* (%)**				
Obesity	55 (31.8)	25 (58.1)	30 (23.1)	<0.001
Hyperlipidemia	35 (20.2)	15 (34.9)	20 (15.4)	0.009
Arterial hypertension	132 (76.3)	36 (85.7)	96 (76.2)	0.19
Chronic kidney disease	31 (17.9)	11 (25.6)	12 (15.4)	0.18
Chronic obstructive pulmonary disease	9 (5.2)	2 (4.8)	7 (5.6)	1.00
**Medication, *n* (%)**				
Aspirin	91 (52.6)	23 (57.5)	68 (56.2)	0.89
Statin	118 (74.2)	28 (70.0)	90 (75.6)	0.48
Beta-blocker	109 (63.0)	27 (69.2)	82 (68.3)	0.92
ACE inhibitor	82 (47.4)	26 (65.0)	56 (47.5)	0.056
**Echocardiographic parameters**				
Ejection fraction, %	60 (55–65)	60 (55–65)	60 (55–65)	0.73
Mean aortic gradient, mmHg	50.3 ± 17.7	60.0 ± 18.8	47.1 ± 16.2	<0.001
Max aortic gradient, mmHg	81.4 ± 27.3	96.7 ± 29.8	76.5 ± 24.7	<0.001
Aortic valve area, cm^2^	0.8 (0.7–1.0)	0.8 (0.6–1.0)	0.8 (0.7–1.0)	0.14
**Routine laboratory investigations**				
Glucose, mmol/L	5.4 (5.0–5.8)	5.4 (5.1–5.8)	5.4 (4.9–5.7)	0.26
Creatinine, µmol/L	79 (67–92)	82 (66–99)	78 (67–88)	0.23
Total cholesterol, mmol/L	4.23 (3.72–5.06)	4.45 (3.96–5.59)	4.18 (3.60–4.92)	0.007
LDL-C, mmol/L	2.41 (1.94–3.24)	2.63 (1.96–3.73)	2.28 (1.93–3.17)	0.11
HDL-C, mmol/L	1.40 (1.17–1.76)	1.41 (1.08–1.79)	1.38 (1.18–1.75)	0.87
Triglycerides, mmol/L	1.16 (0.87–1.68)	1.57 (1.16–1.98)	1.03 (0.78–1.52)	<0.001
Lipoprotein (a), mg/dL	11.9 (4.0–58.2)	16.6 (4.8–91.4)	11.2 (3.6–51.0)	0.11
CRP, mg/L	1.35 (0.90–2.88)	1.52 (1.00–3.00)	1.35 (0.90–2.84)	0.77
**Oxidative stress markers**				
TBARS, nmol/mL	7.76 (6.78–9.03)	7.94 (7.19–9.35)	7.66 (6.63–8.94)	0.11
Plasma PC, nmol/mg	2.76 ± 0.45	2.97 ± 0.45	2.69 ± 0.43	<0.001
**Coagulation**				
Fibrinogen, g/L	3.41 ± 0.73	3.5 ± 0.78	3.38 ± 0.71	0.36
TF, pg/mL	80.9 (64.5–92.8)	82.4 (64.7–90.8)	79.6 (64.3–93.7)	0.69
Prothrombin, %	106.9 ± 15.9	112.64 ± 17.16	104.99 ± 15.10	0.006
**Fibrinolysis proteins**				
Plasminogen, %	97 (89–109)	102 (92–116)	95 (87–107)	0.006
α2-antiplasmin, %	94.5 ± 7.3	98.12 ± 5.85	93.27 ± 7.40	<0.001
PAI-1, ng/mL	11.5 (7.8–16.8)	19.9 (13.7–22.4)	10.0 (7.4–13.7)	<0.001
TAFI, %	81.8 ± 14.1	84.38 ± 15.39	84.38 ± 15.39	0.16
**Fibrin clot properties**				
K_s_ (thrombin-based), 10^−9^ cm^2^	4.39 (3.47–5.59)	4.03 (3.33–5.18)	4.52 (3.50–5.94)	0.14
K_s_ (TF-based), 10^−9^ cm^2^	5.53 (4.57–6.76)	5.42 (4.57–6.45)	5.56 (4.60–6.88)	0.36
CLT, min	91.5 (79.3–103.7)	116.0 (107.6–126.0)	85.5 (77.0–94.7)	<0.001
Lys50, s	472 (401–565)	546 (416–634)	463 (398–537)	0.003

BMI, body mass index; ACE, angiotensin-converting-enzyme; CLT, clot lysis time; LDL, low-density lipoprotein; HDL, high-density lipoprotein; CRP, C-reactive protein; Ks, permeability coefficient; Lys50, lysis time; plasma PC, plasma protein carbonyls; PAI-1, plasminogen activator inhibitor-1, TAFI, thrombin activatable fibrinolysis inhibitor; TBARS, thiobarbituric acid reactive substances, TF, tissue factor.

**Table 2:** Associations between clot lysis time and echocardiographic and laboratory parameters.

Variable	Multivariate *			Multivariate ** (R^2^ = 0.54)		Multivariate *** (R^2^ = 0.55)	
	β (95% CI)	R^2^	*p*-Value	β (95% CI)	*p*-Value	β (95% CI)	*p*-Value
Mean aortic gradient	0.29 (0.14–0.43)	0.15	<0.001	0.22 (0.11–0.33)	<0.001	0.13 (0.02–0.25)	0.027
Plasma PC	11.03 (5.21–16.85)	0.20	<0.001			4.96 (0.25–9.69)	0.039
Prothrombin	0.41 (0.26–0.57)	0.21	<0.001	0.24 (0.08–0.39)	0.003		
α2-antiplasmin	0.82 (0.47–1.17)	0.18	<0.001	0.59 (0.26–0.92)	<0.001	0.69 (0.38–1.00)	<0.001
Plasminogen	0.32 (0.15–0.49)	0.15	<0.001			0.23 (0.09–0.38)	0.001
PAI-1	1.39 (1.06–1.73)	0.34	<0.001	1.25 (0.98–1.52)	<0.001	1.22 (0.92–1.54)	<0.001
Triglycerides	7.11 (3.31–10.90)	0.15	<0.001	4.11 (1.17–7.05)	0.006		
Lipoprotein(a)	0.06 (−0.00–0.12)	0.13	0.051			0.04 (0.00–0.09)	0.045
TAFI	0.07 (−0.12–0.25)	0.08	0.49			−0.11 (−0.25–0.09)	0.11
Maximum aortic gradient	0.17 (0.07–0.27)	0.13	<0.001				
Fibrinogen	4.52 (0.69–8.34)	0.11	0.021				
K_s_	−2.10 (−3.66– −0.53)	0.12	0.01				
Total cholesterol	5.08 (2.23–7.94)	0.14	<0.001				
LDL-C	3.18 (0.27–6.08)	0.10	0.033				

β denotes linear regression estimate. CI, confidence interval. For other abbreviations, see Table 1. * Multivariate model for individual variables adjusted for age and BMI. ** Multivariate model adjusted for age, sex, and BMI. *** Multivariate model adjusted for age, sex, BMI, plasma PC, plasminogen, lipoprotein(a), TAFI, and CRP.

**Table 3:** Predictors of prolonged clot lysis time (CLT) and lysis time (Lys50) defined as the top quartile (≥104 min and >565 s, respectively) in aortic stenosis patients.

Variable	OR per	Univariate *		Multivariate **	
		OR (95% CI)	*p*-Value	OR (95% CI)	*p*-Value
**CLT**					
Mean aortic gradient	1 mmHg	1.05 (1.03–1.07)	<0.001	1.07 (1.03–1.11)	<0.001
Plasma PC	1 nmol/mg	6.41 (2.58–17.83)	<0.001	5.93 (1.42–29.80)	0.021
α2-antiplasmin	1 %	1.10 (1.04–1.17)	0.013	1.18 (1.08–1.30)	<0.001
Plasminogen	1%	1.03 (1.00–1.06)	0.027		
PAI-1	1 ng/mL	1.17 (1.10–1.25)	<0.001	1.18 (1.09–1.28)	<0.001
Triglycerides	1 mmol/L	2.89 (1.63–5.39)	<0.001	7.09 (2.75–22.06)	<0.001
**Lys50**					
Plasma PC	1 nmol/mg	5.83 (2.23–15.21)	<0.001	7.61 (2.44–23.70)	<0.001
TBARS	1 μM	1.34 (1.11–1.63)	0.003		
α2-antiplasmin	1 %	1.16 (1.08–1.25)	<0.001	1.11 (1.02–1.20)	0.013
Fibrinogen	1 g/L	3.09 (1.68–5.69)	<0.001	2.49 (1.16–5.37)	0.020
Prothrombin	1 %	1.07 (1.03–1.10)	<0.001		

OR, odds ratio; CI, confidence interval. For other abbreviations, see Table 1. * Adjusted for age, sex, and BMI. ** Multivariate models were fitted using backward stepwise regression and adjusted for age, sex, and BMI.
